# What's the bleeding problem: menstrual health and living with a disability

**DOI:** 10.1016/j.lanwpc.2021.100349

**Published:** 2021-12-22

**Authors:** Penelope A. Phillips-Howard

**Affiliations:** Department of Clinical Sciences, Liverpool School of Tropical Medicine, Pembroke Place, Liverpool L3 5QA

**Keywords:** MHH, Menstrual health and hygiene, LMIC, Low- and middle-income countries, WASH, Water, hygiene and sanitation

## Commentary

Menstrual health and hygiene (MHH) is defined as having safe and easy access to the information, supplies, and infrastructure needed to manage menstruation with dignity and comfort (menstrual hygiene management) and also the systemic factors that link menstruation with health, gender equality, empowerment, and beyond.^1^ Despite some 1.9b individuals experiencing menstruation worldwide, it remain a neglected topic of research and public health, with the World Bank estimating some 500 million do not attain menstrual health.^2^^,^^8^ A systematic review of studies noted menstruation remains a negative experience with stigma, shame and secrecy associated with menstruating, made more complicated by a lack of information, education, or support.^3^

Layered upon this, consider the challenges and difficulties faced by the 110-190 million people with disabilities,^4^ including those with intellectual impairments, who rely on informal or professional caregivers for assistance.^5^ Such persons require the education, social environment, products and materials, and water hygiene and sanitation (WASH) facilities, plus additional support to overcome the challenges and barriers they face due to their disability. In a prior review, Wilber and colleagues highlighted the many deficits in good practice ranging from a lack of standard guidance for caregivers, or menstrual training, information and support for those with intellectual impairments.^6^ They further noted few data were available on understanding the symptoms experienced by people with intellectual impairments, and that menstrual products were expensive and lacked appropriate options for people with physical impairments.^6^

While menstrual health is increasingly recognized as a public health issue that is linked with human rights and attainment of the sustainable development goals,^1^^,^^7^ it is still not recognised as a priority for all.^8^ Action has focused largely on schoolgirls and humanitarian settings, and provision of menstrual products to schoolgirls.^7^ Attention is thus needed to support policy and guidance on menstrual needs across the life course and among particular vulnerable populations isolated from view. Resources beyond menstrual products such as information, water and sanitation facilities, other supplies, and menstruation-friendly environments are equally needed.^8^

In this volume of The Lancet Regional Health - Western Pacific, Wilbur and colleagues now report on a detailed mixed method study, which has examined the experiences and needs of menstruators with disabilities in Vanuatu against current best practice for menstrual health.^9^ Compared with others in the household, those with disabilities had a three to five times higher odds of using different bathing facilities, missing social activities while menstruating, and isolated eating. Difficulties were encountered with managing materials, cleaning and collecting water, increasing stigma and impacting on comfort, safety, hygiene.

Wilbur and colleagues systematic review emphasized the pitifully small number of studies conducted among disabled persons and their menstrual needs; of 22 worldwide, research was predominantly in high income countries; three were from India, and one each from Malawi, south Africa and Turkey, representing the voices of just a few hundred persons with disabilities and caregivers across low- and middle income countries (LMIC).^6^ This current study^9^ provides a platform for broader and deeper enquiry into the experiences of those with disabilities living in differing geographies and settings in LMIC. By employing multiple methodologies including a census, nested case-control study, in-depth interviews, focus group discussions, photovoice, and structured discussions, the study provides an exemplar on how to collect the breadth of contextual data among a carefully selected representative sample.^10^ Such depth of knowledge and methodological rigor add great value to guide research in menstrual health, which has largely focused on descriptive studies.^11^ The comparative methodology ensures insight into similarities and differences in experiences and needs between those with and without disabilities, in urban and rural settings.

The authors recognise the findings may not be generalizable due to cultural differences, and thus replication of this research is required in other settings. The findings reveal clear evidence that knowledge-oriented interventions and supportive devices to improve menstrual health need to be developed and tested. This includes materials that dispel harmful social beliefs and taboos that discriminate against all who menstruate, but even more profoundly affect those with disabilities. An attempt to quantify a ‘menstruation interference score’ found those with no disability reported greater daily interference from menstruation, although those with disabilities suffered greater restrictions. Alternative indicators to measure intervention effects and quality of life evaluation are thus needed. Studies around costs of interventions, benefits, and return on investment are required to inform implementation strategies and policy frameworks. Additionally, Wilber and colleagues prior review identified evidence that forced cessation of menstruation through hysterectomies occurs among disabled, and warrants urgent attention.^5^

## Key messages

• Persons with disabilities suffer greater disadvantages in care and support of their menstruation.
• Caregivers lack knowledge-based guidance and supportive devices which deleteriously impacts adequate menstrual health and hygiene practices.
• Interventions are urgently needed to provide dignity and care and ameliorate social stigma and taboos which increase isolation and restrictions among those with disabilities.

## Declaration of interests

None.
